# Supplementary material for: Increased Cytokine Levels in Seronegative Myositis: Potential Th17 Immune Response Implications
Source: Int J Mol Sci. 2024 Oct 15;25(20):11061. doi: 10.3390/ijms252011061 (PMC11508411; doi:10.3390/ijms252011061)
Supplement: Supplementary file 1 [file ijms-25-11061-s001.zip › Supplementary Table 1.pdf]

Table 4. Cytokine and chemokine serum levels of MII patients according to treatment.

Cytokine/ Chemokine (pg/ml)	Immunosuppressive Treatment								
	Prednisone			Azathioprine			Hydroxychloroquine		
	Yes	No	*P*[#]	Yes	No	*P*[#]	Yes	No	*P*[#]
IL-1β, med (IQR)	0.0 (0.0 – 89.9)	2.3 (0.0 – 115.7)	0.733	0.0 (0.0 – 9.6)	1.7 (0.0 – 115.7)	0.527	3.5 (0.0 – 65.3)	0.0 (0.0 – 115.7)	0.820
IFN-α2, med (IQR)	3.7 (0.0 – 93.0)	2.1 (0.0 – 156.9)	0.651	5.3 (0.9 – 7.2)	3.6 (0.0 – 156.9)	0.805	1.4 (0.0 – 93.0)	3.8 (0.0 – 157.9)	0.117
IFN-γ, med (IQR)	0.0 (0.0 – 131.3)	3.6 (0.0 – 157.9)	0.514	0.0 (0.0 – 0.0)	4.4 (0.0 – 157.9)	0.163	4.9 (0.0 – 131.3)	0.0 (0.0 – 157.9)	0.213
TNF-α, med (IQR)	0.0 (0.0 – 1741.3)	10.3 (0.0 – 418.6)	0.442	0.0 (0.0 – 4.1)	0.0 (0.0 – 1741.3)	0.629	0.0 (0.0 – 1741.3)	0.0 (0.0 – 418.6)	0.565
IL-6, med (IQR)	10.5 (0.0 – 621.9)	24.6 (0.0 – 139.2)	0.390	0.0 (0.0 – 2.4)	12.7 (0.0 – 621.9)	**0.010**	24.5 (2.4 – 621.9)	6.0 (0.0 – 139.2)	0.114
IL-10, med (IQR)	2.7 (0.0 – 195.7)	7.7 (0.0 – 99.5)	0.579	1.7 (0.0 – 3.7)	4.7 (0.0 – 195.7)	0.359	3.7 (0.0 – 195.7)	1.7 (0.0 -99.5)	0.923
IL-12p70, med (IQR)	0.0 (0.0 – 98.5)	8.7 (0.0 – 74.5)	0.266	0.0 (0.0 – 0.6)	0.0 (0.0 – 98.5)	0.629	0.0 (0.0 – 98.5)	0.0 (0.0 – 74.5)	0.941
IL-17A, med (IQR)	0.2 (0.0 – 33.4)	4.1 (0.4 – 32.0)	**0.028**	0.0 (0.0 – 0.5)	1.0 (0.0 – 33.4)	0.116	0.6 (0.0 – 33.4)	0.6 (0.0 – 32.0)	0.459
IL-18, med (IQR)	521.34 (0.0 – 12157.9)	469.0 (75.8 – 1300.7)	0.452	579.8 (0.0 – 3915.0)	462.9 (75.8 – 12157.9)	0.935	591.8 (128.2 – 12157.9)	435.6 (0.0 – 3915.0)	0.228
IL-23, med (IQR)	1.5 (0.0 – 282.4)	10.4 (0.0 – 505.2)	0.236	1.2 (0.0 – 282.4)	3.3 (0.0 – 505.2)	0.870	7.6 (0.0 – 244.5)	1.4 (0.0 – 505.2)	0.184
IL-33, med (IQR)	0.0 (0.0 – 1979.9)	232.1 (0.0 – 2530.3)	**0.018**	0.0 (0.0 – 0.0)	0.0 (0.0 – 2530.3)	0.261	0.0 (0.0 – 1979.9)	0.0 (0.0 – 2530.3)	0.864
CCL2, med (IQR)	249.5 (42.4 – 1022.5)	160.2 (55.8 – 480.8)	0.787	259.4 (104.1 – 818.5)	241.5 (42.4 – 1022.5)	0.452	78.9 (43.5 – 343.7)	266.5 (42.4 – 1022.5)	**0.026**
CXCL8, med (IQR)	3.5 (0.0 – 1096.1)	61.1 (0.0 – 655.3)	0.118	4.2 (0.0 – 13.6)	7.0 (0.0 – 621.9)	0.450	7.0 (0.0 – 1096.12)	6.7 (0.0 – 655.3)	0.954

IL-1β: interleukin-1β; **IFN-α2:** interferon- α2; **IFN-γ:** interferon-γ; **TNF-α:** tumor necrosis factor-α; **IL-6:** interleukin-6; **IL-10:** interleukin-10; **IL-12p70:** interleukin-12p70; **IL-17A:** interleukin-17A; **IL-18:** interleukin-18; **IL-23:** interleukin-23; **IL-33:** interleukin-33; **CCL2:** chemokine (C–C motif) ligand 2; **CXCL8:** chemokine (C-X-C motif) ligand 8. [#]Kruskal-Wallis test with Monte Carlo test.
