# Supplementary figures and images for: Increased Cytokine Levels in Seronegative Myositis: Potential Th17 Immune Response Implications
Source: Int J Mol Sci. 2024 Oct 15;25(20):11061. doi: 10.3390/ijms252011061 (PMC11508411; doi:10.3390/ijms252011061)

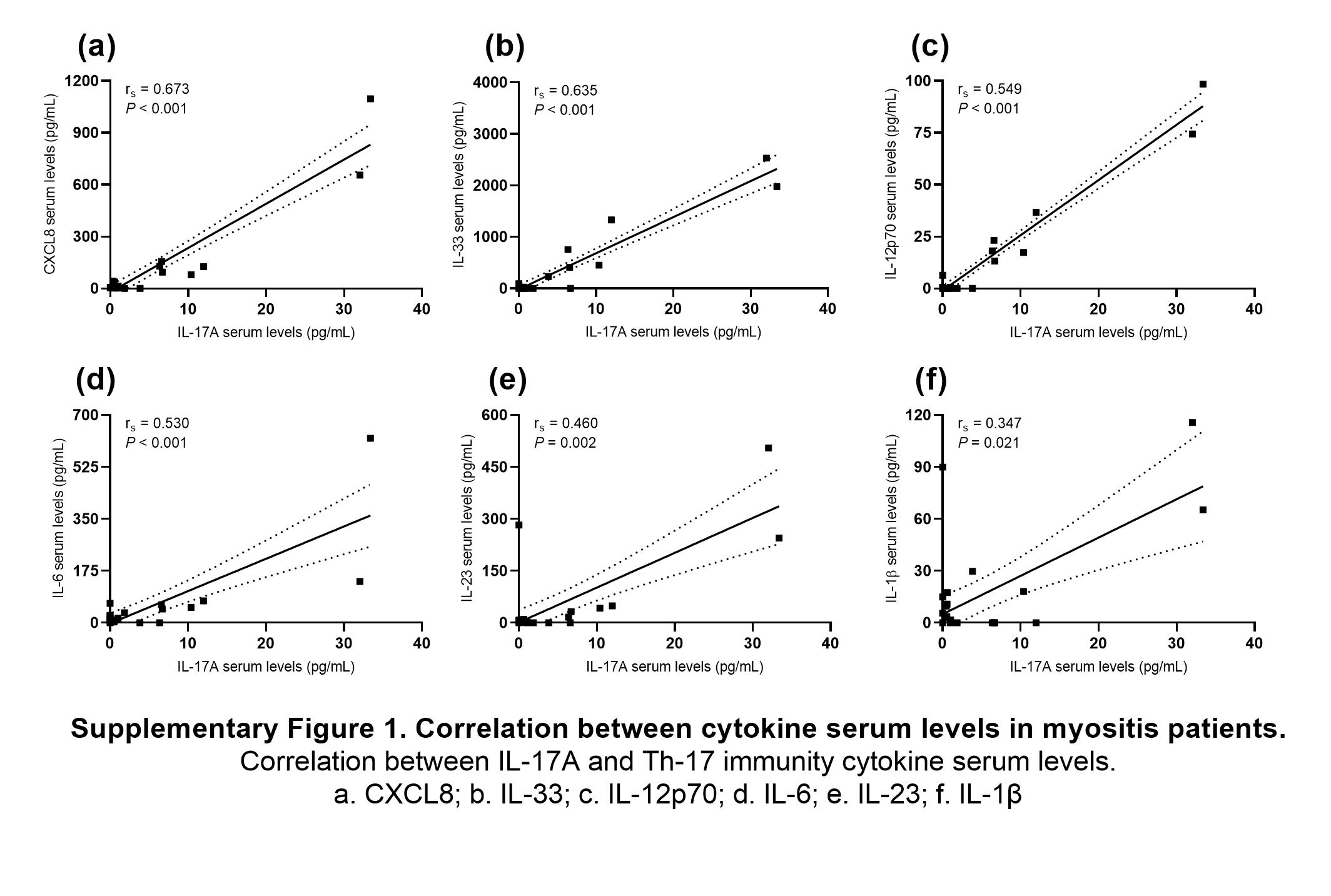

Supplement: Supplementary file 1 [file ijms-25-11061-s001.zip › Supplementary Figure 1.jpg]
